# The Treatment of Pruritus Pudendi

**Published:** 1887-05

**Authors:** E. S. McKee

**Affiliations:** Cincinnati, Ohio


### THE TREATMENT OF PRURITUS PUDENDI.

BY E. S. MCKEE, M. D., OF CINCINNATI, OHIO.

[Extract of a Paper read before the Cincinnati Academy of Medicine.]

First we should ascertain the cause of disease, to treat it intelligently. We should treat the constitutional disease, the origin of the trouble ; next we should treat the morbid phenomenon, the pruritus. Remove the cause and the pruritus will disappear of itself. The parts should be washed twice a day with Castile soap and water ; the diet should be vegetable, and regular action of the bowels maintained ; as a general rule, stimulants should be disallowed. In this troublesome trouble—for we can scarcely call it a disease—we need all the remedies we can find, hence I give all I know :

4-per cent. solution of boracic acid.
2–10-per cent. solution of carbolic acid.
3–5-per cent. solution of silver nitrate.
0–5-per cent. solution of bi-chloride of mercury.
25–50-per cent. solution of sulphurous acid.
6-per cent. solution of sodium bi-borate.
Ointments of tar, boracic acid, camphor or iodoform.
Mixtures of camphor and chloral.
Infusions of tobacco.

20-per cent. solution of chloroform in almond oil.

Treatment with the bi-chloride should be preceded by a removal of the mucus with warm water and the parts then dried with a soft linen. Pass a sponge, moistened with the solution, rapidly over the affected parts. This leaves a burning, smarting sensation, which is alleviated by a few minutes' washing with cold water. Subsequent applications become less and less painful.

M. Dubois recommends, in the rebellious cases, that the entire surface of the vulva be cauterized with a solid stick of the nitrate of silver. The great objection to this is that it is extremely painful and the alleviation produced by it is almost always temporary.

Meigs recommends—

℞. Sodii biboratis..............................ʒ ij,
Morph. sulph.............................gr. ivss,
Aquæ rosæ dest.........................fl. ℥ viij. M.

Sig. Apply three times a day to the affected part, with a sponge or soft piece of linen. Take care to wash well the parts beforehand with soap and warm water, and dry well afterward. A compress dipped in the oil of sweet almonds and laid in the commissure of the vagina is recommended.

When the trouble is general, temporary relief may be obtained by placing the woman in a prolonged soda bath, and subsequently rubbing the entire surface with vaseline.

Pruritus, which has extended upon the distended abdominal walls, is well treated with—

℞. Lin. saponis comp..............................f ℥ v,

Chloroformi..................................f ℥ j.

Sig. Apply locally.

If the itching comes from an ulcerated cervix or, more properly, from the irritating discharge proceeding from it, apply nitrate of silver and introduce tampon of tanno-glycerine.

Pruritus from breeding pediucli is well treated by mild mercurial ointments. Stavesacre answers well. A plasm formed of flowers of sulphur and water; saline purgatives, as Pullna or Friederichshall water, Vichy baths, or even bathing with cold or tepid water constitute the best purgatives. Salines and colchicum may be indicated, also bromide of potassium. A weak solution of Goulard's lotion, or lotion of—

℞. Liq. morphinæ hydrochlor......................f ℥ j,
Acid hydrocyanic dil.........................f ʒ iss,
Aquæ.........................................f ℥ vi. M.

Sig. Use as a lotion.

Pledgets soaked in the following, and placed in the vagina, have been found useful:

℞. Sodii biboratis...........................
Acidi sulphurici dilut.......................
Glycerini, aa. ..............................f ℥ ij. M.

Insert at bedtime and withdraw in the morning.

Iodoform may be dusted over the parts.

The following has often given relief:

℞. Chloroformi............................f ʒ ij,

Ol. amygdal............................f ℥ ij. M.

Sig. Apply externally.

Morphine and chloral, internally, may be found necessary to obtain relief at night. Hildebrant has found the tinct. cannabis indicæ, gtt. 10–20, to be of even more service than these.

There is no end to remedies; the trouble is to get the right one.

℞. Extract opii............................gr. v,
Plumbi acet............................gr. xx,
Acidi hydrocyanici dil............................f ʒ j,
Aquam, ad............................f ℥ j. M.

Sig. Apply on lint to the vulva; or—

℞. Liq. plumbi subacetat............................f ℥ j,

Acidi hydrocyanici dil............................f ʒ j,

Aquam, ad............................Oj.

℞. Acidi tannici............................ʒ ij,

Extracti belladonnæ............................gr. x,

Butyr. cacao............................ʒ v. M.

Div. in suppos. xx.

Sig. Insert one in the vagina, night and morning.

℞. Sodii biborat............................ʒ ij,

Morph. sulph............................gr. vj,

Aquæ rosæ............................f ℥ viij. M.

Sig. Apply to the vulva on lint.

Trousseau recommends—

℞. Potassii carbonat............................ʒ ij,

Aquæ............................f ℥ iv. M.

Sig. Lotion.

℞. Hydrargyri chloridi mitis............................ʒ j,

Aquæ............................f ℥ j. M.

Ft. unguent.

Sig. Apply locally.

A solution of nitrate of silver (20 grains to the fluidounce), applied to the neck and cervical canal so far as accessible, will often remove the pruritus even when due to pregnancy. If due to vesicular eruptions on the genitals this application should be made to the affected part.

Fox recommends the following:

℞. Sodii hyposulphitis............................ʒ iv,

Glycerini............................f ʒ ij,

Aquam dest, ad............................f ℥ vi. M.

Sig. Lotion.

℞. Hydrargyri bichloridi............................gr. j,
Acidi hydrocyanici dil............................f ʒ j,
Emuls. amygdal............................f ℥ vj.

℞. Acid hydrocyanici dil........................f ʒ ss,

Infus. althæa..............................f ℥ v. M.

Sig. Apply daily.

℞. Sodii biborat.............................ʒ j,

Acid hydrocyanici dil.......................f ʒ ij,

Aquæ rosæ.... ..............................f ʒ viij. M.

Sig. Use in the pruritus of old people.

℞. Ammonii acet..............................℥ j,

Acid hydrocyanici dil.......................f ʒ iss,

Infus. nicotiani............................f ℥ viij. M.

Sig. Apply twice, daily.

McCall Anderson recommends :

℞. Potassii cyanidi..........................gr. vj,

Pulv. cocci............. ....................gr. j,

Unguent, aquæ rosæ..........................℥ j. M.

Sig. Use as ointment.

M. Gueneau de Mussy recommends the following :

℞. Infus. althæa.........................1 litre,
Aquæ laurocerasi.........................50.00 grammes,
Sodii borat..............................20.00 grammes. M.

He then prescribes an ointment, to be used night and morning, as follows :

℞. Glycerole of starch...................20.00 grammes,
Potassium bromide.......................
Bismuth subnitrate, aa.................. 1.00 grammes,
Calomel............................... 0.40 grammes. M.

De Savignac uses the above lotion, then dusts the surface with the following powder :

℞. Pulv. lycopodii.......................30.00 grammes,
Bismuthi subnitratis.....................19.00 grammes,
Bellad. rad.... ......................... 2.00 grammes. M.

Dr. Martineau recommends an ointment of cocaine, 1 in 10.

It is claimed by German writers that this pruritus is localized on a certain small area of the mucous membrane, and that by the removal of this part by the knife we remove the cause. This, I think, would have no effect on a case dependent upon the pregnant state, a discharge, or the condition of the urine.—*Clinical Record.*

